# Capacity for Seeding and Spreading of Argyrophilic Grain Disease in a Wild-Type Murine Model; Comparisons With Primary Age-Related Tauopathy

**DOI:** 10.3389/fnmol.2020.00101

**Published:** 2020-06-24

**Authors:** Isidro Ferrer, Pol Andrés-Benito, Julia Sala-Jarque, Vanessa Gil, José Antonio del Rio

**Affiliations:** ^1^Department of Pathology and Experimental Therapeutics, University of Barcelona, Barcelona, Spain; ^2^Bellvitge University Hospital, IDIBELL (Bellvitge Biomedical Research Centre), Barcelona, Spain; ^3^CIBERNED (Network Centre of Biomedical Research of Neurodegenerative Diseases), Institute of Health Carlos III, Ministry of Economy and Competitiveness, Madrid, Spain; ^4^Institute of Neurosciences, University of Barcelona, Barcelona, Spain; ^5^Molecular and Cellular Neurobiotechnology, Institute of Bioengineering of Catalonia (IBEC), Institute for Science and Technology, Parc Científic de Barcelona, Barcelona, Spain; ^6^Department of Cell Biology, Physiology and Immunology, Faculty of Biology, University of Barcelona, Barcelona, Spain

**Keywords:** argyrophilic grain disease, primary age-related tauopathy, tauopathies, tau, seeding, progression, coiled bodies

## Abstract

Argyrophilic grain disease (AGD) is a common 4R-tauopathy, causing or contributing to cognitive impairment in the elderly. AGD is characterized neuropathologically by pre-tangles in neurons, dendritic swellings called grains, threads, thorn-shaped astrocytes, and coiled bodies in oligodendrocytes in the limbic system. AGD has a characteristic pattern progressively involving the entorhinal cortex, amygdala, hippocampus, dentate gyrus, presubiculum, subiculum, hypothalamic nuclei, temporal cortex, and neocortex and brainstem, thus suggesting that argyrophilic grain pathology is a natural model of tau propagation. One series of WT mice was unilaterally inoculated in the hippocampus with sarkosyl-insoluble and sarkosyl-soluble fractions from “pure” AGD at the age of 3 or 7/12 months and killed 3 or 7 months later. Abnormal hyper-phosphorylated tau deposits were found in ipsilateral hippocampal neurons, grains (dots) in the hippocampus, and threads, dots and coiled bodies in the fimbria, as well as the ipsilateral and contralateral corpus callosum. The extension of lesions was wider in animals surviving 7 months compared with those surviving 3 months. Astrocytic inclusions were not observed at any time. Tau deposits were mainly composed of 4Rtau, but also 3Rtau. For comparative purposes, another series of WT mice was inoculated with sarkosyl-insoluble fractions from primary age-related tauopathy (PART), a pure neuronal neurofibrillary tangle 3Rtau + 4Rtau tauopathy involving the deep temporal cortex and limbic system. Abnormal hyper-phosphorylated tau deposits were found in neurons in the ipsilateral hippocampus, coiled bodies and threads in the fimbria, and the ipsilateral and contralateral corpus callosum, which extended with time along the anterior-posterior axis and distant regions such as hypothalamic nuclei and nuclei of the septum when comparing mice surviving 7 months with mice surviving 3 months. Astrocytic inclusions were not observed. Tau deposits were mainly composed of 4Rtau and 3Rtau. These results show the capacity for seeding and spreading of AGD tau and PART tau in the brain of WT mouse, and suggest that characteristics of host tau, in addition to those of inoculated tau, are key to identifying commonalities and differences between human tauopathies and corresponding murine models.

## Introduction

Argyrophilic grain disease (AGD) is a neurodegenerative disorder morphologically characterized by the accumulation of 4R hyper-phosphorylated tau protein in dendritic swellings known as argyrophilic grains, neurons with pre-tangles, coiled bodies in oligodendrocytes, and astrocytes with the morphology of thorn-shaped astrocytes (TSAs), located predominantly in limbic regions of the brain (Braak and Braak, 1987, 1989; Tolnay et al., 1997; Botez et al., 1999; Togo et al., 2002; Zhukareva et al., 2002; Tolnay and Clavaguera, 2004; Ferrer et al., 2008; Tolnay and Probst, 2008; Tolnay and Braak, 2011; Kovacs, 2015; Ikeda et al., 2018; Rösler et al., 2019). Ballooned neurons containing αB-crystallin are also constant in the amygdala in AGD (Tolnay and Probst, 1998).

The frequency of AGD increases with age; it can be clinically silent or manifested by progressive cognitive impairment and dementia which makes it difficult to distinguish from Alzheimer’s disease (AD) (Braak and Braak, 1989; Jellinger, 1998; Ding et al., 2006; Rodriguez and Grinberg, 2015). Moreover, AGD is commonly associated with AD, other tauopathies, Lewy body diseases, and TDP-43 proteinopathy (Braak and Braak, 1989; Ferrer et al., 2008; Josephs et al., 2008; Fujishiro et al., 2009; Kovacs et al., 2013; Yokota et al., 2018). The majority of cases of AGD are sporadic but a few cases have been linked to mutations in the *MAPT* gene (Kovacs et al., 2008; Rönnbäck et al., 2014).

Neuropathological studies of routine series suggest that AGD shows an archetypal pattern of progression (Saito et al., 2004; Ferrer et al., 2008). AGD stage 1 affects the anterior entorhinal cortex, part of the cortical and basolateral nuclei of the amygdala, and the hypothalamic lateral tuberal nucleus; stage 2 involves a greater number of lesions and progression to the whole entorhinal cortex, anterior CA1, transentorhinal cortex, cortical and basolateral nuclei of the amygdala, presubiculum, hypothalamic lateral tuberal nucleus, and dentate gyrus; stage 3 further involves CA1, perirhinal cortex, presubiculum, amygdala, dentate gyrus, hypothalamic lateral tuberal nucleus, CA2 and CA3, subiculum, other nuclei of the hypothalamus including the mammillary bodies, anterior temporal cortex, insular cortex, anterior cingulated gyrus, orbitofrontal cortex, nucleus accumbens, and septal nuclei; and stage 4 is characterized by further moderate-to-severe involvement of the neocortex and brainstem (Saito et al., 2004; Ferrer et al., 2008; Tolnay and Probst, 2008). This pattern has prompted consideration of argyrophilic grain pathology as a natural model of tau propagation (Clavaguera et al., 2013a; Rábano et al., 2014). Indeed, inoculation of brain homogenates from AGD cases to the brain of mice transgenic for wild-type human tau (line ALZ17) leads to the capacity for abnormal tau seeding and propagation (Clavaguera et al., 2013b, 2015). In addition to tau deposits in neurons and threads, phospho-tau immunoreactivity is seen in oligodendrocytes forming coiled bodies, and in astrocytes reminiscent of TSAs, following injection of AGD homogenates in the hippocampus and in the cerebral cortex (Clavaguera et al., 2013a). Moreover, brain extracts from AGD patients have the capacity to transmit tau to HEK293 cells expressing 4Rtau, thus suggesting that prion-like tau strains can also propagate in cultured cells (Woerman et al., 2016).

Our previous studies have shown tau seeding and spreading following inoculation of sarkosyl-insoluble fractions from AD, primary age-related tauopathy (PART), aging-related tau astrogliopathy (ARTAG), progressive supranuclear palsy (PSP), Pick’s disease (PiD), frontotemporal lobar degeneration linked to *MAPT* P301L mutation, and sporadic and familial globular glial tauopathy (GGT) into the hippocampus and corpus callosum of WT mice (Ferrer et al., 2018, 2019, 2020a,b). The present study is focused on the capacity for and characteristics of seeding and propagation of phospho-tau from homogenates of “pure” (not associated with AD or other tauopathies) AGD cases in the hippocampus of WT mice. For comparative purposes, another set of mice was unilaterally inoculated in the hippocampus with sakosyl-insoluble and -soluble fractions of PART, a 3Rtau + 4Rtau pure neuronal tauopathy with neurofibrillary tangles (Crary et al., 2014), that is considered part of AD (Duyckaerts et al., 2015).

## Materials and Methods

### Human Brain Samples

Brain samples of the hippocampus were obtained from the Institute of Neuropathology Brain Bank, Bellvitge University Hospital, following the guidelines of the Spanish legislation on this matter (Real Decreto Biobancos 1716/2011), and the approval of the local ethics committee of the Bellvitge University Hospital (Hospitalet de Llobregat, Barcelona, Spain). The agonal state was short with no evidence of acidosis or prolonged hypoxia; the pH of each brain was between 6.8 and 7. At the time of autopsy, one hemisphere was fixed in paraformaldehyde for no less than 3 weeks and selected brain sections were embedded in paraffin; de-waxed paraffin sections, 4 microns thick, were processed with neuropathological and immunohistochemical methods as detailed elsewhere (Ferrer, 2014). The other hemisphere was cut into coronal sections 1 mm thick, and selected brain regions were dissected, immediately frozen at −80°C, put on labeled plastic bags, and stored at −80°C until use; the rest of the coronal sections were frozen and stored at −80°C (Ferrer, 2014). Four AGD cases were used for this study, all of them men, aged 63, 67, 74, and 75 years old. The four cases were categorized as stage 3 of AGD. Two cases had pre-tangles, threads and grains but not NFTs; one of them had NFT stage I and another NFT stage II following Braak and Braak staging of AD-related pathology. β-amyloid deposits were absent in every case. Other AGD cases from our series with marked AD pathology were excluded from the present study. AGD cases with other tauopathies such as PSP and corticobasal degeneration, and cases with concomitant TDP-43 proteinopathy or with Lewy body disease, were excluded. This selection permitted a reasonable approach to the study of “pure” AGD pathology.

Two PART cases (one man aged 68 and one woman aged 72) were selected on the basis of pure neurofibrillary tangle (NFT) pathology with no accompanying tauopathy and without β-amyloid deposits (Crary et al., 2014). NFT burden in PART cases was categorized as stage IV according to Braak and Braak modified for paraffin sections (Braak and Braak, 1991; Braak et al., 2006). The subjects did not have apparent neurological deficits and the diagnosis was made at the post-mortem neuropathological examination.

One case (a 65-year-old man) with no neurological disease and with no lesions on neuropathological examination, and one case of AD stage V-VI/C (one woman aged 82) were used as negative and positive controls, respectively.

### Western Blotting

Frozen samples of the hippocampus from the four AGD and the two PART cases were processed in parallel with one AD case (Braak and Braak stage V–VI/C) and one case with no lesions. Frozen samples of about 1 g were lysed in 10 volumes (w/v) with cold suspension buffer (10 mM Tris-HCl, pH 7.4, 0.8 M NaCl, 1 mM EGTA) supplemented with 10% sucrose, protease, and phosphatase inhibitors (Roche, GE). The homogenates were first centrifuged at 20,000 × g for 20 min (Ultracentrifuge Beckman with 70Ti rotor) and the supernatant (S1) was saved. The pellet was re-homogenized in five volumes of homogenization buffer and re-centrifuged at 20,000 × g for 20 min. The two supernatants (S1 + S2) were then mixed and incubated with 0.1% N-lauroylsarkosynate (sarkosyl) for 1 h at room temperature while being shaken. Samples were then centrifuged at 100,000 × g for 1 h. Sarkosyl-insoluble pellets (P3) were re-suspended (0.2 ml/g) in 50 mM Tris–HCl (pH 7.4). Protein concentrations were quantified with the bicinchoninic acid assay (BCA) assay (Pierce, Waltham, MA, United States).

Sarkosyl-insoluble and sarkosyl-soluble fractions were processed for western blotting.

Samples were mixed with loading sample buffer and heated at 95°C for 5 min. 60 μg of protein was separated by electrophoresis in SDS-PAGE gels and transferred to nitrocellulose membranes (200 mA per membrane, 90 min). The membranes were blocked for 1 h at room temperature with 5% non-fat milk in TBS containing 0.2% Tween and were then incubated with the primary phospho-specific antibody anti-tau Ser422 (diluted 1:1,000; Thermo Fisher Scientific (Waltham, MA, United States). After washing with TBS-T, blots were incubated with the appropriate secondary antibody (anti-rabbit IgG conjugated with horseradish peroxidase diluted at 1:2,000, Agilent, United States) for 45 min at room temperature. Immune complexes were revealed by incubating the membranes with chemiluminescence reagent (Amersham, GE Healthcare, Life Sciences, Buckinghamshire, United Kingdom) (Ferrer et al., 2018).

### Thioflavin T (ThT) Amyloid Quantification Assay

ThT stock solution was prepared at 2.5 mM (dissolved in 10 mM phosphate buffer, 150 mM NaCl, pH 7.0) and preserved in single-aliquot at −80°C. The ThT assay was performed by dissolving 0.2 μL of sarkosyl-insoluble fraction in 0.2 mL of freshly prepared ThT (final concentration 30 μM) followed by quantification using an absorbance/excitation (445/485) microplate reader (Tecan Infinite M200Pro, Männedorf, Switzerland) in 96 flat bottom polystyrol plates (Nunclor, Thermo Fisher Scientific, GE). Plates were prepared and incubated at 37°C. Readings were taken each hour over the course of 15–16 h.

### Animals and Brain Inoculation

Wild-type C57BL/6 mice from our colony were used. All animal procedures were carried out following the guidelines of the European Communities Council Directive 2010/63/EU and with the approval of the local ethical committee (C.E.E.A: Comitè Ètic d’Experimentació Animal; University of Barcelona, Spain; ref. 426/18).

One series of mice was inoculated with sarkosyl-insoluble fractions of a mixture of the two AGD cases with no NFTs, in which western blotting of sarkosyl-insoluble fractions showed only the bands of 68 and 64 kDa typical of 4Rtaupathies. The animals were inoculated at the age of 3 months and killed at the age of 6 months (*n* = 2, survival 3 months); 3 months and killed at the age of 10 months (*n* = 1, survival 7 months); 7 months and killed at the age of 10 months (*n* = 2, survival 3 months); and 12 months and killed at the age of 19 months (*n* = 2, survival 7 months). Two mice were inoculated with a mixture of sarkosyl-soluble fractions at the age of 7 months and killed at the age of 10 months (*n* = 2, survival 3 months); and one mouse was inoculated with vehicle (50 mM Tris-HCl, pH 7.4) at the age of 3 months and killed at the age of 10 months (survival 7 months). Three additional inoculated mice died shortly after anesthesia and were not included in the study.

For comparative purposes, another series of mice was inoculated with sarkosyl-insoluble fractions of two PART cases at the age of 3 months and killed at the age of 10 months (*n* = 3, survival 7 months); 7 months and killed at the age of 10 months (*n* = 3, survival 3 months); 12 months and killed at 19 months (*n* = 4, survival 7 months). Finally, mice were inoculated with PART sarkosyl-soluble fractions at the age of 7 months and killed at the age of 10 months (*n* = 2, 3 months survival); and with control brain homogenates at the age of 3 or 7 months and killed at the age of 10 months (*n* = 2, 7 and 3 months survival, respectively). The total number of available mice considering the two series was 24.

Mice were deeply anesthetized by intra-peritoneal ketamin/xylazine/buprenorphine cocktail injection and placed in a stereotaxic frame after assuring lack of reflexes. Intra-cerebral injections were administered using a Hamilton syringe; the coordinates for hippocampal injections were −1.9 mm AP; −1.4 mm ML relative to Bregma (interaural 1.8 mm), and −1.5 mm DV from the dural surface (Paxinos and Franklin, 2019). A volume of 1.5 μL was injected at a rate of 0.05 μL/min. The syringe was withdrawn slowly over a period of 10 min to avoid leakage of the inoculum. Following surgery, the animals were kept in a warm blanket and monitored until they recovered from the anesthesia. Carprofen analgesia was administered immediately after surgery and once a day during the next 2 consecutive days. Animals were housed individually with full access to food and water.

### Tissue Processing

Animals were killed under anesthesia and the brains were rapidly fixed with 4% paraformaldehyde in phosphate buffer, and embedded in paraffin. Consecutive serial sections 4 μm thick were obtained with a sliding microtome. De-waxed sections were stained with hematoxylin and eosin, Congo Red, and Thioflavin T, or processed for immunohistochemistry using the antibodies anti-phospho-tau Thr181, anti-phospho-tau Ser262, Ser422, AT8 (Ser202/Thr205), PHF1(Ser396/Ser404) (donation of Dr. Peter Davies, Albert Einstein College of Medicine, Bronx, NY, United States), anti-4Rtau, anti-3Rtau, and tau-C3 (tau truncated at aspartic acid 421), in addition to GFAP for reactive astrocytes and Iba1 for microglia. The characteristics of the antibodies are shown in Table 1. Following incubation with the primary antibody, the sections were incubated with EnVision + system peroxidase for 30 min at room temperature. The peroxidase reaction was visualized with diaminobenzidine and H_2_O_2_. Control of the immunostaining included omission of the primary antibody; no signal was obtained following incubation with only the secondary antibody. The specificity of 3Rtau and 4Rtau antibodies in mice was tested in coronal sections of the brain, cerebellum, and brainstem of P301S transgenic mice aged 8–9 months old (López-González et al., 2015). Tau-immunoreactive inclusions in animals expressing mutant human 4Rtau were stained with anti-4Rtau antibodies but they were negative with anti-3Rtau antibodies.

**TABLE 1:** Characteristics and dilutions of the antibodies used for immuno-histochemistry and immunofluorescence.

Antibody	Mono-/polyclonal	Dilution	Supplier	Country
4Rtau	Monoclonal	1:50	Merck-Millipore	Billerica, MA, United States
3Rtau	Monoclonal	1:800	Merck-Millipore	Billerica, MA, United States
Phospho-tau Thr181	Rabbit polyclonal	1:50	Cell Signaling	Danvers, MA, United States
Phospho-tau Ser262	Rabbit polyclonal	1:100	Calbiochem-Merck	Kenilworth, NJ, United States
Phospho-tau Ser422	Rabbit polyclonal	1:200	Thermo Fisher Scientific	Waltham, MA, United States
AT8 (Ser202/Thr205)	Monoclonal	1:50	Innogenetics	Ghent, BE
PHF1(Ser396/Ser404)	Monoclonal	1:20	Dr. Peter Davies	New York, United States
Tau-C3 (tr Asp421)	Monoclonal	1:300	Abcam	Cambridge, United Kingdom
P38-P (Thr180-Tyr182)	Rabbit polyclonal	1:100	Cell Signaling	Danvers, MA, United States
Glial fibrillary acidic protein (GFAP)	Rabbit polyclonal	1:500	Dako	Glostrup, DK
Iba1	Rabbit polyclonal	1:1000	Wako	Richmond, VA, United States
Olig2	Rabbit polyclonal	1:500	Abcam	Cambridge, United Kingdom
NeuN	Monoclonal	1:100	Merck-Millipore	Billerica, MA, United States

Double-labeling immunofluorescence was carried out on de-waxed sections, 4 μm thick. The sections were boiled in citrate buffer to enhance antigenicity and blocked for 30 min at room temperature with 10% fetal bovine serum diluted in 0.1 M phosphate-buffered saline (PBS). The sections were stained with a saturated solution of Sudan black B (Merck, DE) for 15 min to block autofluorescence of putative lipofuscin granules present in cell bodies, and then rinsed in 70% ethanol and washed in distilled water. Then, the sections were incubated at 4°C overnight with combinations of AT8 and one of the following primary antibodies: GFAP, Iba-1, Olig2, and p38-P (Thr180-Tyr182). Other sections were immunostained with anti-phospho-tauThr181 and anti-NeuN (Table 1). After washing, the sections were incubated with Alexa488 or Alexa546 fluorescence secondary antibodies against the corresponding host species. Nuclei were stained with DRAQ5^TM^. Then the sections were mounted in Immuno-Fluore^TM^ mounting medium (MP Biomedicals, CA, United States), sealed, and dried overnight. Sections were examined with a Leica TCS-SL confocal microscope.

*In situ* end-labeling of nuclear DNA fragmentation (ApoptTag^TM^ peroxidase *in situ* apoptosis detection kit, Merck, GE) was used to visualize apoptotic cells. The brains of newborn irradiated rats (2Gys) with a survival time of 24 h were fixed with paraformaldehyde and embedded in paraffin; de-waxed sections were processed in parallel with tissue samples from inoculated mice and used as positive controls of apoptosis.

### Semi-Quantitative Studies

Since lesions in mice inoculated with sarkosyl-insoluble fractions of AGD surviving 3 and 7 months were similar, and the same occurred following inoculation of PART (see later), the total number of mice for each time of survival was considered for semi-quantitative studies as follows: AGD 3 months, *n* = 4, AGD 7 months, *n* = 3; PART 3 months, *n* = 2, PART, 7 months *n* = 7. Quantification of neurons and oligodendrocytes was made in an area of 0.35 μm^2^ in affected regions in every case. Symbols indicate the average number of labeled cells: neurons: –:0, #: 1, ##: 2–3; ###: >3; oligodendrocytes: –: 0, ϕ: 1–3, ϕϕ: 4–7, ϕϕϕ: >7. Regarding grains, threads and dots, values were expressed as –:0, +: mild; ++: moderate; and +++: abundant.

### Quantitative Studies

The number of neurons in the hippocampus, oligodendrocytes in the ipsilateral corpus callosum, and oligodendrocytes in the contralateral corpus callosum was counted in three different fields of 0.035 μm^2^ in affected regions per mouse at the different survival times (3 and 7 months) in the four groups of mice (AGD and PART). Counts were compared with two-way analysis of variance (ANOVA) followed by Tukey’s *post-hoc* test using SPSS software. Differences were considered statistically significant at ^∗∗^*P* < 0.01 and ^∗∗∗^*P* < 0.001 compared with AGD 3 months group; $$*P* < 0.01 and $$$*P* < 0.001 compared with AGD 7 months group; and ###*P* < 0.001 compared with PART 3 months group. Comparison between values of oligodendrocytes in ipsilateral (ipsi) and contralateral (contr) corpus callosum within the same PHFs type and incubation time were assessed (comparison of two regions at the same period of time) using T-student test and SPSS software. Differences were considered statistically significant at ^xx^*P* < 0.001 compared with AGD 3 months oligo. Ipsi group; &&&*P* < 0.001 compared with AGD 7 months oligo. Ipsi group; ^^^*P* < 0.001 compared with PART 3 months oligo ipsi group; and +++*P* < 0.001 compared with PART 7 months oligo ipsi group. Outliers were detected using the GraphPad software QuickCalcs (*P* < 0.05). Statistical analysis and graphic design were performed with GraphPad Prism version 5.01 (La Jolla, CA, United States).

## Results

### AGD in the Human Hippocampal Complex

The four cases were categorized as stage 3 of AGD. Two cases had pre-tangles, threads, and grains, but not NFTs. The other two cases had, in addition, some NFTs in the entorhinal cortex and very few in the hippocampus.

Pre-tangles were localized in the pre-subiculum, subiculum, CA2, and CA3 areas of the hippocampus, and granule cells in the dentate gyrus. Large numbers of grains were localized in the pre-subiculum (in addition to the entorhinal and trans-entorhinal cortices), and CA1 region of the hippocampus. Threads, which were characterized as fine neuritic processes, were present in the same areas as grains, and in the white matter of the hippocampus and entorhinal region.

Oligodendroglial inclusions, mainly with the morphology of coiled bodies, were found in the hippocampal white matter. TSAs were found in the periventricular white matter, and sparsely in the white matter of the inner region of the temporal lobe.

**FIGURE 1:**
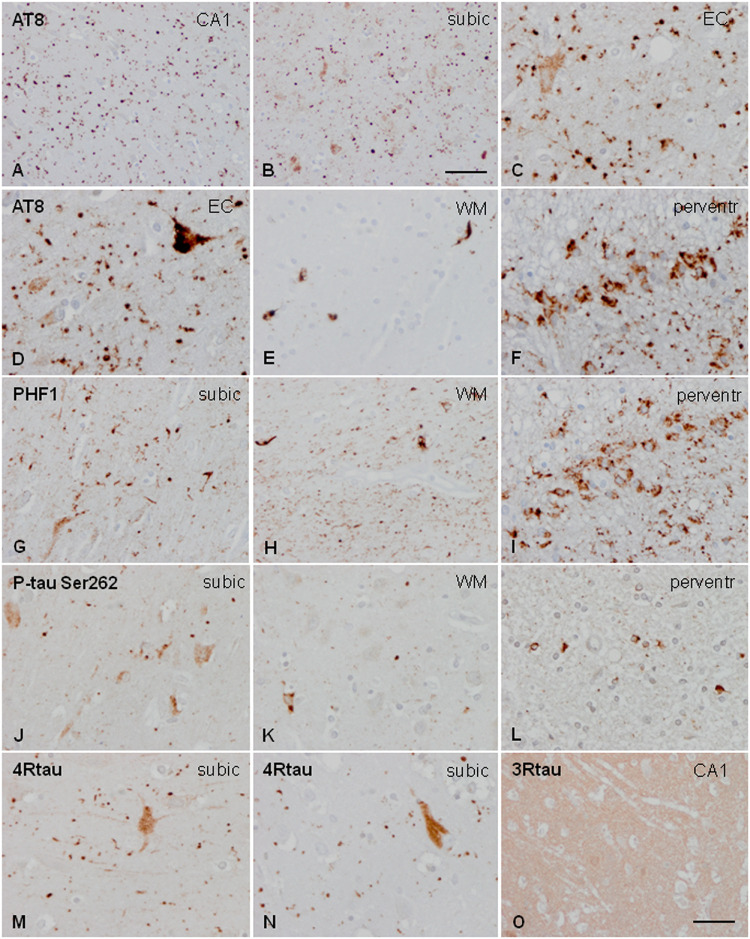
Characteristics of tau deposits in AGD. Hyper-phosphorylated tau deposits are seen in the CA1 region of the hippocampus (CA1: **A**), subiculum (subic: **B,F,J,M,N**), entorhinal cortex (EC: **C**), white matter of the hippocampus (WM: **E,H,K**), and periventricular region of the temporal horn (perventr: **F,I,L**). Grains are identified as small round, lobulated, or comma-shaped deposits in the neuropil **(A–C,D,G,J)**. Grains are accompanied by pre-tangles in neurons **(C,D,G,J)**, and, rarely, by neurons with dense granular inclusions **(D)**. In addition, oligodendroglial deposits form coiled bodies and cytoplasmic inclusions around the nucleus **(E,H,K)**. Thorn-shaped astrocytes (TSAs) are common in the periventricular white matter **(F,I,L)**. All these deposits are stained with AT8 antibodies **(A–F)**, PHF1 **(G–I)**, and anti-P-tau Ser262 **(J–L)**. Grains and pre-tangles are composed of 4Rtau **(M,N)** and do not contain 3Rtau **(O)**. Paraffin sections, processed for immunohistochemistry, and slightly counterstained with hematoxylin; **(A,B)**, bar = 55 μm; **(D–O)**, bar = 30 μm.

Neuronal, astrocytic, and oligodendroglial inclusions, as well as grains and threads, were labeled with anti-phospho-tau antibodies Thr181, Ser262, Ser422, AT8, and PHF1. All these deposits were positive with ant-4Rtau antibodies and negative with anti-3Rtau antibodies (excepting very few oligodendrocytes) (Figure 1).

Tau-C3 immunoreactivity was restricted to NFTs and isolated coiled bodies.

**FIGURE 2:**
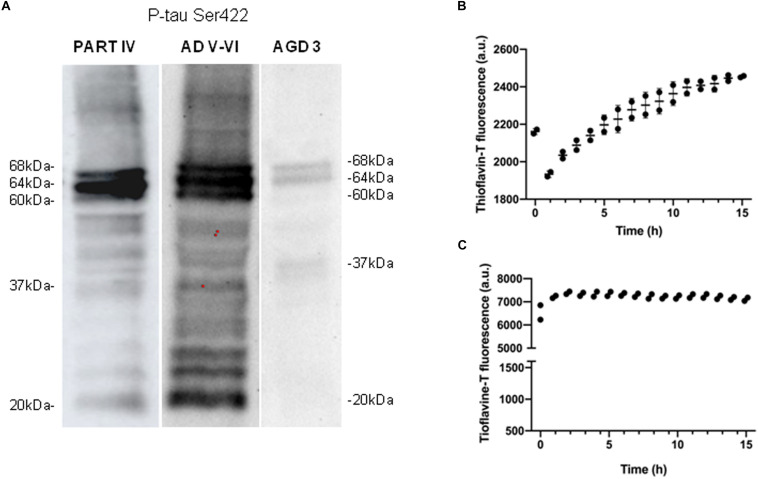
Characteristics of sarkosyl-insoluble fractions. Western blotting processed with anti-phospho-tau Ser422 (P-tau Ser422) in PART stage IV and AD stages V–VI reveals three bands of 68, 64, and 60 kDa, and upper oligomeric smears, in addition to several bands of about 50 kDa, between 30 and 40 kDa, and lower bands of truncated tau at the C-terminal, one of them of about 20 kDa. In contrast, “pure” AGD reveals two bands of 68 and 64 kDa, and a weak band of about 37 kDa **(A)**. Thioflavin T incubation of sarkosyl-insoluble fractions shows slightly increased fluorescence with time indicating moderate fibril formation in AGD up to 2,500 au **(B)**, and rapid and higher fluorescence in PART reaching about 7,500 au up to 15 h of incubation **(C)**.

Western blotting of sarkosyl-insoluble fractions processed with anti-phospho-tau Ser422 revealed two bands of 68 and 64 kDa, and a weak band of about 37 kDa in AGD cases. This pattern was different from that seen in one AD stage V-VI/C processed in parallel which was characterized by three bands of 68, 64, and 60 kDa, together with several bands of about 50 kDa, several bands between 30 and 40 kDa, and lower bands of truncated tau at the C-terminal, one of which was about 20 kDa, in addition to oligomeric species of higher molecular weight (Figure 2A). The same pattern was observed in all four cases, although a lower band of truncated tau was noted in the two cases categorized as AGD plus NFT stage II after long exposure (30 min). Incubation of sarkosyl-insoluble fraction with thioflavin showed moderately increased fluorescence with time (Figure 2B).

### PART in the Human Hippocampal Complex

PART cases were characterized by numerous NFTs in the entorhinal, transentorhinal cortex, presubiculum, subiculum, hippocampus (with preservation of the dentate gyrus), and temporal cortex. Threads were also abundant in these regions, but dystrophic neurites were absent. No tau deposits were found in astrocytes or oligodendrocytes. The morphology and distribution of NFTs corresponded to stage IV of Braak and Braak, linked to AD pathology. β-amyloid deposits were absent.

NFTs were identified by their typical flame-like morphology in pyramidal neurons which were composed of 4Rtau and 3Rtau, and stained with several specific anti-phospho-tau antibodies: Thr181, Ser262, Ser422, AT8, and PHF1. NFTs were also positive with antibody tau-C3 which is raised against truncated tau at aspartic acid 421. This is exactly the same profile seen in AD (Ferrer et al., 2013).

As in AD, PART cases were characterized by three bands of 68, 64, and 60 kDa, and several lower bands of 50 kDa and between 30 and 40 kDa, together with strong lower bands stained with anti-tauSer422 indicating truncated tau at the C-terminal. Oligomeric species of higher molecular weight wee manifested as smear. This pattern is typical of 4Rtau + 3Rtau tauopathies and identical to that seen in AD (Figure 2A).

ThT amyloid quantification assay of sarkosyl-insoluble fractions revealed positive curves of amyloid fibrils. Interestingly, fluorescence appeared earlier and the intensity was higher in PART than in AGD (Figure 2C).

### AGD Inoculation in the Hippocampus of WT Mice

**FIGURE 3:**
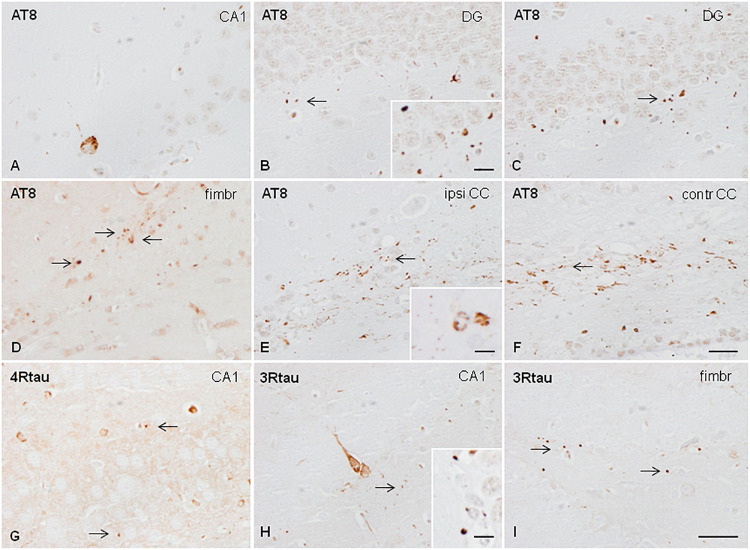
Hyper-phosphorylated tau-containing cells and threads following unilateral hippocampal injection of sarkosyl-insoluble fractions from AGD in WT mice inoculated at 7 months and killed at the age of 10 months (3 months of survival) **(A,B)**; inoculated at the age of 3 months and killed at the age of 6 months (3 months survival) **(C)**; inoculated at the age of 12 months and killed at the age of 19 months (7 months survival) **(D–F)**; and inoculated at the age of 3 months and killed at the age of 10 months (survival 7 months) **(E–H)**. Neurons in the ipsilateral hippocampus contain granular hyper-phosphorylated tau deposits as revealed with the AT8 antibody without morphological features of tangles **(A)**; these deposits are stained with antibodies against 4Rtau and 3Rtau **(G,H)**. Grains (dots) in CA1 region of the hippocampus, dentate gyrus, fimbria, and corpus callosum are recognized with the antibody AT8 **(B,C,E,F)**, anti P-tau Ser262 **(D)**, and antibodies against 4Rtau and 3Rtau **(G–I)** (thin arrows). Threads and coiled bodies containing hyper-phosphorylated tau are seen in the ipsilateral and contralateral corpus callosum in mice surviving 7 months after inoculation. Paraffin sections slightly counterstained with hematoxylin. CA1, region of the hippocampus; DG, dentate gyrus; fimbr, fimbria; ipsi contr CC, ipsi- and contralateral corpus callosum; **(A–F)**, bar = 25 μm; **(G–I)**, bar = 25 μm. Grains, coiled bodies and grains, insets in **(A,E,H)**, respectively, bar = 7.5 μm.

A mixture of sarkosyl-insoluble fractions from the two cases without associated NFT pathology was inoculated into the hippocampus of WT mice at the age of 3 or 7 months. Mice were killed, respectively, at the age of 6 or 10 months (3 months of survival; *n* = 2 for every condition). Inoculation resulted in the deposition of hyper-phosphorylated tau in neurons of the CA1 region of the hippocampus and dentate gyrus, threads in the fimbria and corpus callosum, oligodendoglial deposits in the fimbria and ipsilateral corpus callosum, and grains (dots) in the dentate gyrus, fimbria, and corpus callosum. Neuronal tau deposition consisted of diffuse or fine granular deposits; neurofibrillary tangles were not seen in any case at any time. Oligodendroglial tau aggregates had the morphology of perinuclear deposits or of coiled bodies. Deposits were immunostained with anti-phospho-tau antibodies raised against single specific phospho-tau sites Thr181 and Ser262, double-phosphorylated sites as revealed with antibodies AT8 (Ser202/Thr205), and PHF1 (Ser396/Ser404); and with antibodies raised against 4Rtau and 3Rtau (Figure 3). Immunoreactivity to tau-C3 was largely negative excepting a few grains and threads in the corpus callosum. Mice inoculated at the age of 3 or 12 months, and killed at the age of 10 or 19 months (7 months of survival; *n* = 1 and *n* = 2, respectively), showed, in addition to the previous deposits, an extension of lesions to the contralateral corpus callosum characterized by threads, dots, and coiled bodies (Figure 3). No tau deposits were seen in other brain regions. No differences were seen between the animals inoculated Tau deposits were negative with Congo Red, and did not show fluorescence with thioflavin T. All the animals inoculated with sarkosyl-insoluble fractions showed tau deposits.

Similar changes were seen in mice surviving 3 or 7 months despite the age of inoculation.

### WT Mice Inoculated With Sarkosyl-Insoluble Fractions From PART

**FIGURE 4:**
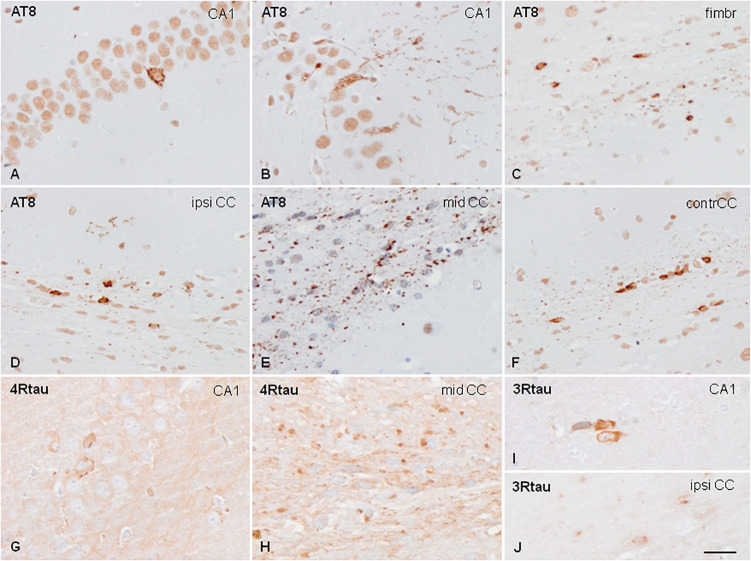
Hyper-phosphorylated tau-containing cells and threads following unilateral intra-hippocampal injection of sarkosyl-insoluble fractions from PART into WT mice at the age of 7 months and killed at the age of 10 months (3 months survival) **(A,C)**; 3 months and killed at the age of 10 months **(C,D–F)**; and at the age of 12 months and killed at the age of 19 months (7 months survival) **(G–J)**. Tau deposits in neurons, independently of the survival time, show granular deposits in the cytoplasm, and occasional denser inclusions with no similarities with tangles **(A,B)**. Threads and coiled bodies are abundant in the fimbria and corpus callosum **(C–F)**. Individual neurons, threads and oligodendrocytes in inoculated mice are stained with anti-4Rtau **(G,H)** and anti-3Rtau **(I,J)** antibodies. Paraffin sections slightly counterstained with hematoxylin. CA1, region of the hippocampus; fimbr, fimbria; ipsi contr CC, ipsi- and contralateral corpus callosum; **(A–F)**, bar = 50 μm; **(G–J)**, bar = 50 μm.

Sarkosyl-insoluble fractions of two PART cases were unilaterally inoculated in the hippocampus at the age of 7 months and killed at the age of 10 months (3 months survival); 3 months and killed at the age of 10 months or at the age of 12 months and killed at the age of 19 months (7 months survival). Mice with survival of 3 months showed tau deposits in the ipsilateral hippocampus, fimbria, and ipsilateral corpus callosum extending along the anterior-posterior axis. Mice surviving 7 months, independently of the age of inoculation, showed, in addition to the previous regions, major extension along the anterior-posterior axis, together with tau deposits in the middle and contralateral corpus callosum, the ipsilateral periventricular hypothalamus, and septal nuclei. Tau deposits were found in neurons, threads, and oligodendroglial cells (mainly round and coiled bodies) which were stained with specific anti-phospho tau antibodies Thr181 and Ser262, double-phosphorylated sites as revealed with antibodies AT8 (Ser202/Thr205) and PHF1 (Ser396/Ser404), and with antibodies raised against 4Rtau and 3Rtau (Figure 4). Immunoreactivity to tau-C3 was largely negative excepting a few threads in the corpus callosum. Tau deposits were negative with Congo Red and they did not show fluorescence with thioflavin T.

Mice inoculated with sarkosyl-soluble fractions and those inoculated with control brain homogenates did not show tau deposits.

**FIGURE 5:**
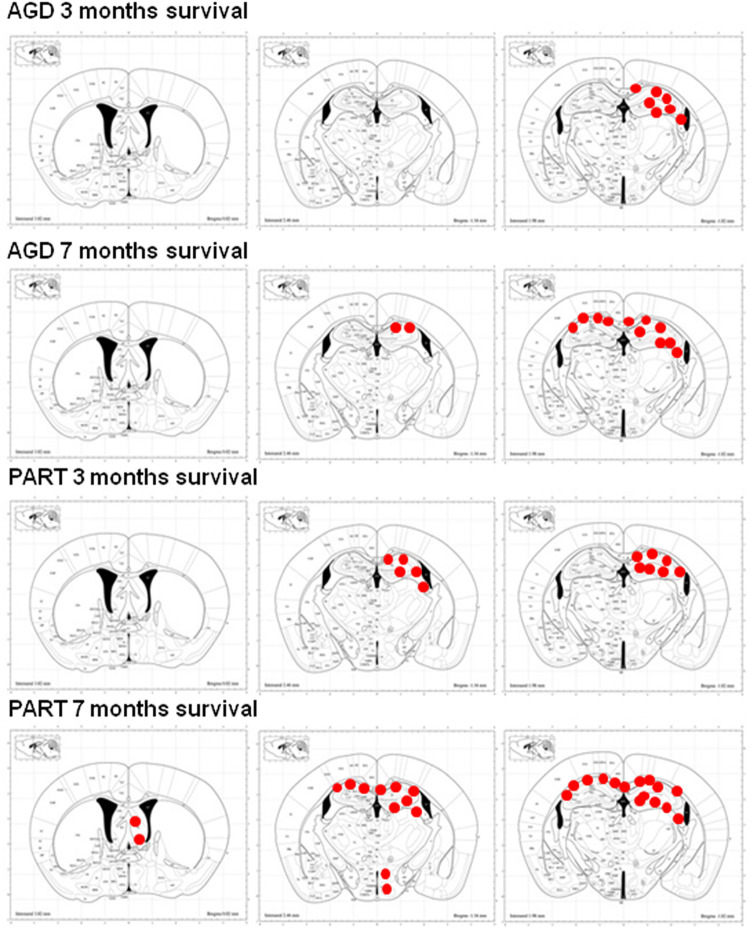
Schematic representation of phospho-tau-immunoreactive deposits (red dots), as revealed with the AT8 antibody, following unilateral inoculation of sarkosyl-insoluble fractions from AGD and PART into the hippocampus of WT mice with survival times of 3 and 7 months. Mice inoculated with AGD and killed 3 months after inoculation show tau deposits restricted to the ipsilateral hemisphere, whereas mice inoculated with PART and killed after 3 months show wider distribution of tau deposits along the anterior-posterior axis although restricted to the same hemisphere. Mice inoculated with AGD and killed 7 months later have, in addition, tau deposits along the contralateral corpus callosum, whereas mice inoculated with PART and killed at 7 months post-inoculation show, in addition to the previous regions, further extension along the anterior-posterior axis, deposits in distal projections such as the periventricular hypothalamus and septal nuclei, and abundant deposits in the contralateral corpus callosum. Maps obtained from the atlas of Paxinos and Franklin. Red dots simply represent the distribution of lesions; no attempt is made to distinguish between deposits in neurons, oligodendrocytes, threads and dots, and quantify the numbers or densities of each one of these lesions.

Similar changes were seen in mice surviving 3 or 7 months despite the age of inoculation. Therefore, the distribution of tau-immunoreactive deposits following inoculation of PART sarkosyl-insoluble fractions with survival times of 3 and 7 months are shown in Figure 5, together with the distribution of deposits of AGD to facilitate comparison. Semi-quantitative studies are summarized in Table 2.

**TABLE 2:** Semi-quantitative representation of lesions following inoculation of sarkosyl-insoluble fractions of AGD and PART at survival times of 3 months (AGD, *n* = 4; PART, *n* = 2) and 7 months (AGD, *n* = 3; PART, *n* = 7).

	Hip neur/gr	fim olig/thr	cc ips olig/thr/dots	cc contr olig/thr/dots	Ant/post axis	Other distant regions
AGD 3 months	##/+	ϕ/++	ϕ/++/+	–	–	–
AGD 7 months	##/+	ϕϕ/++	ϕϕ/+++/++	ϕ/++/+	+	–
PART 3 monts	###/–	ϕϕϕ/++	ϕϕϕ/++/+	–	++	–
PART 7 months	###/–	ϕϕ/++	ϕϕϕ/+++/++	ϕϕϕ/+++/++	+++	§§

*Hip, hippocampus; fim, fimbria; cc ip and contr, corpus callosum ipsilateral and contralateral; ant/post axis, extension along the anterior/posterior axis; other distant regions include nuclei of the hypothalamus and septal nuclei. Symbols per area of 0.035 μm^2^; neurons: –: 0, #: 1, ##: 2–3; ###: >3; oligodendrocytes: –: 0, : ϕ1–3, ϕϕ: 4–7, ϕϕϕ: >7; grains, threads and dots: –:0, +: mild, ++: moderate, +++: abundant; other distant regions (hypothalamus and septal nuclei) –: no; §§: yes.*

**FIGURE 6:**
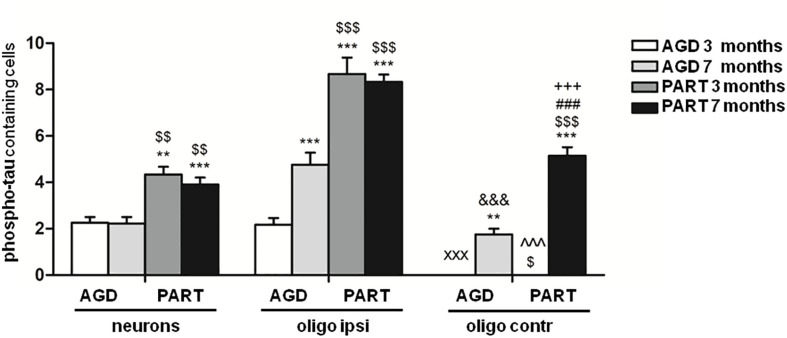
Numbers of neurons in the hippocampus, oligodendrocytes in the ipsilateral corpus callosum, and oligodendrocytes in the contralateral corpus in the four groups of mice inoculated with sarkosyl-insoluble fractions of AGD and PART with 3 months and 7 months of survival. Counts were compared with two-way analysis of variance (ANOVA) followed by Tukey post-test using SPSS software. Differences are considered statistically significant at **P < 0.01 and ***P < 0.001 compared with AGD 3 months; ^$^*P* < 0.05, ^$$^*P* < 0.01, and ^$$$^*P* < 0.001 compared with AGD 7 months; and ^###^*P* < 0.001 compared with PART 3 months. Comparison between values of oligodendrocytes in ipsilateral (ipsi) and contralateral (contr) corpus callosum was assessed using T-student test and SPSS software. Differences are considered statistically significant at ^xxx^*P* < 0.001 compared with AGD 3 months ipsi; ^&&&^*P* < 0.001 compared with AGD 7 months ipsi; ^^^*P* < 0.001 compared with PART 3 months ipsi; and ^+++^*P* < 0.001 compared with PART 7 months ipsi.

To analyze in more detail differences in the number of phospho-tau-containing neurons in the hippocampus in the two groups of mice inoculated with sarkosyl-insoluble fractions of AGD and PART with different incubation times, we performed a statistical study using a two-way ANOVA test followed by Tukey’s *post-hoc* test. Two-way ANOVA did not reveal significant interactions between the type of the inoculums (AGD or PART) and the survival time [*F*(1, 44) = 0.31, *p* = 0.57]. Subsequent Tukey’s *post-hoc* test showed significant differences between group AGD 3 months compared with group PART 3 months (*P* = 0.003), and group PART 7 months (*P* = 0.001), but not with the group AGD 7 months. Mice AGD 7 months showed reduced number of positive neurons when compared with mice PART 3 months (*P* = 0.005) and PART 7 months (*P* = 0.003). Regarding the number of oligodendroglial cells in the ipsilateral corpus callosum between groups, two-way ANOVA revealed a significant interaction between the inoculated PHF type and the incubation time in phosphor-tau-immunoractive oligodendroglial cells [*F*(1, 43) = 10.41, *p* = 0.0024]. Differences were significant when comparing AGD and PART (PHF type) [*F*(1, 43) = 124.4 *p* < 0.0001] and the survival time (3 vs. 7 months) [*F*(1, 43) = 6.19, *p* = 0.017]. *Post-hoc* test showed significant increase in phospho-tau containing oligodendroglial cells in the ipsilateral corpus callosum in AGD 7 months (*P* = 0.001), PART 3 months (*P* = 0.000), and PART 7 months (*P* = 0.000) when compared with AGD 3 months. Similarly, significant increase in the number of phospho-tau containing oligodendrocytes was noticed in PART 3 months (*P* = 0.000) and PART 7 months (*P* = 0.000) when compared with AGD 7 months. Regarding phospho-tau containing oligodendroglial cells in the contralateral corpus callosum, two-way ANOVA revealed significant interaction between the type of inoculums (AGD and PART) and the survival time [*F*(1, 47) = 21.25, *p* < 0.0001]. Significant differences were found in relation with the effects of the inoculums (AGD or PART) [*F*(1, 47) = 21.25 *p* < 0.0001] and the survival time (3 or 7 months) [*F*(1, 47) = 87.72, *p* < 0.0001]. Tukey’s *post-hoc* test revealed significant increased numbers of phospho-tau immunoreactive oligodendroglial cells in contralateral corpus callosum in mice of the groups AGD 7 months (*P* = 0.004) and PART 7 months (*P* = 0.000) when compared with mice of the AGD 3 months group. Following the same trend, increased numbers of phospho-tau containing oligodendrocytes in the contralateral corpus callosum was seen in the groups AGD 7 months and PART 7 months when compared with PART 3 months (*P* = 0.016 and *P* = 0.000, respectively). These values show that the number of labeled neurons in the hippocampus, and oligodendroglial cells in the ipsilateral and contralateral callosum was significantly higher in mice inoculated with PART sarkosyl-insoluble fractions when compared with mice inoculatyed with AGD fractions (Figure 6).

Finally, the number of phospho-tau-positive oligodendrocytes in the ipsilateral corpus callosum was compared with the number of phospho-tau-positive oligodendrocytes in the contralateral corpus callosum at the same survival time in every one of the four groups. Significant differences were seen between the ipsilateral corpus callosum and the contralateral corpus callosum in mice AGD 3 months (*P* = 0.000); AGD 7 months *P* = 0.000); PART 3 months (*P* = 0.000); and PART 7 months (*P* = 0.000). These values indicated significant progression of phospho-tau deposits in oligodendrocytes in the contralateral corpus callosum with time after inoculation (Figure 6).

*In situ* end-labeling of nuclear fragmentation was negative in mice inoculated with sarkosyl-insoluble fractions from AGD and PART processed in parallel with brain sections of newborn irradiated rats killed 24 h after irradiation (2Gy) (data not shown).

No hyper-phosphorylated tau deposition occurred in mice inoculated with sarkosyl-soluble fractions or with vehicle alone (data not shown). 4Rtau and 3Rtau deposits were also absent in these mice used as controls.

### Double-Labeling Immunofluorescence and Confocal Microscopy

**FIGURE 7:**
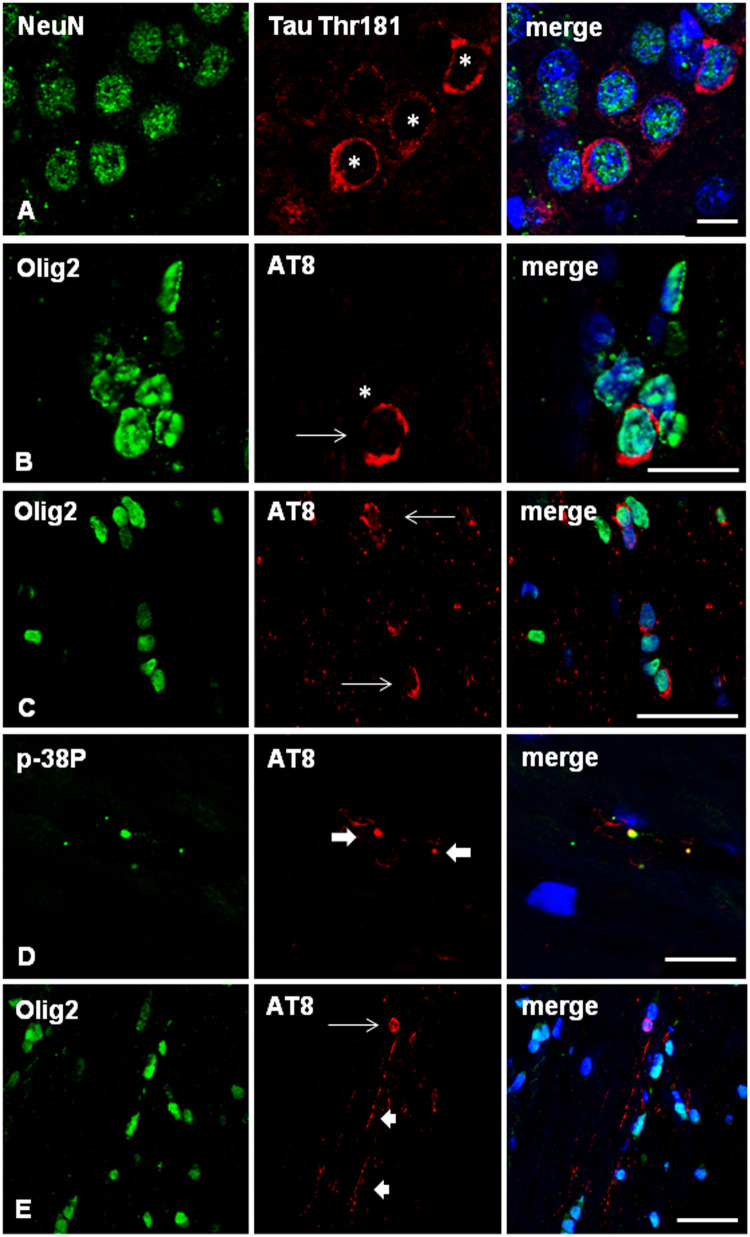
**(A–D)** WT mice unilaterally inoculated in the hippocampus with sarkosyl-insoluble fractions from AGD at the age of 12 months and killed at 19 months (7 months survival) showing tau-immunofluorescent CA1 neurons (asterisks) in the ipsilateral hippocampus **(A)**, and deposits of hyper-phosphorylated tau in the cytoplasm of oligodendrocytes (**B,C**; arrows) as revealed with double-labeling immunofluorescence to NeuN (green) and P-tauThr181 (red), and Olig2 (green) and AT8 (red), respectively. Double-labeling immunofluorescence to phosphorylated p38 (p38-P, p38-PThr180-182) (green) and AT8 (red) in WT mice inoculated with sarkosyl-insoluble fractions from AGD at the age of 12 months and killed at the age of 19 months (7 months survival) **(D)**. Active p38 kinase (p38-P) co-localizes with tau deposits in tau-positive grains (thick arrows) and threads in the hippocampus. E: WT mouse unilaterally inoculated in the hippocampus with sarkosyl-insoluble fractions from PART at the age of 12 months and killed at 19 months (7 months survival) showing tau-immunofluorescent oligodendrocyte (thin arrow) and threads (short arrows) in the contralateral corpus callosum. Paraffin sections, nuclei stained with DRAQ5^TM^ (blue); **(A)**, bar = 10 μm; **(B)**, bar = 30 μm; **(C,D)**, bar = 10 μm; **(E)** = 25 μm.

Localization of phospho-tau in neurons and oligodendrocytes in AGD-inoculated mice was identified with double-labeling immunofluorescence with anti-phospho-tau antibodies in combination with NeuN and Olig2 antibodies, respectively (Figures 7A–C). Antibodies anti-p38-P (Thr180-Tyr182) revealed co-localization with phospho-tau (AT8) in the majority of grains or dots (Figure 7D), as well as in pre-tangles and coiled bodies, as detailed in other tauopathies (Ferrer et al., 2018, 2019, 2020a).

Double-labeling immunofluorescence to GFAP and Iba1, and to AT8, confirmed the absence of phospho-tau deposits in astrocytes and microglia, respectively, at all times during the study.

Similar features regarding neurons and glial cells were assessed in PART-inoculated mice using double-labeling immunofluorescence and confocal microscopy. In addition to neurons, oligodendrocytes and threads were easily visualized in the corpus callosum (Figure 7E). As for AGD, no astrocytes or microglia contained phospho-tau deposits.

## Discussion

Tau seeding and spreading occur following unilateral inoculation of sarkosyl-insoluble fractions from AD, PART, ARTAG, PSP, PiD, FTLD linked to *MAPT* P301L mutation, and GGT in the hippocampus of WT mice using the protocol utilized in the present study (Ferrer et al., 2018, 2019, 2020a,b). Neurons and oligodendrocytes are the main targets of tau spreading, which progresses through synaptically connected areas, and along tracts such as the corpus callosum to reach the contralateral hemisphere in those settings. The present results show a similar pattern of tau seeding and spreading following unilateral inoculation of sarkosyl-insoluble fractions from homogenates of AGD cases without concomitant tauopathy, and more particularly without NFTs, in the hippocampus of WT mice. Neurons and oligodendrocytes are the main targets; in addition, grains (dots) and threads containing phospho-tau are present in the hippocampus and in the corpus callosum. The morphological characteristics of tau deposits in neurons, as revealed with phospho-specific anti-tau antibodies, are similar to those seen in human AGD cases bearing only pre-tangles; threads and dots are also similar to those seen in AGD, but their presence in the corpus callosum indicates that most “grains” in inoculated mice are not located in dendrites, in contrast to the ordinary dendritic localization of grains in AGD (Ferrer et al., 2008). Therefore, what we call grains in AGD-inoculated mice are probably small aggregates of phospho-tau in neuronal processes other than dendrites. Coiled bodies in AGD-inoculated mice are the counterparts of coiled bodies in AGD. Yet astrocytes do not contain phospho-tau in inoculated mice at the survival times (3 and 7 months) assessed in the present study, in contrast with the presence of TSAs in AGD. The characteristics of tau as revealed by immunohistochemistry are similar in AGD and in AGD-inoculated WT mice. Of note, the presence of P-tauSer262 in the inclusions a phosphorylation is also seen in AGD (Ferrer et al., 2002). Co-localization of phosphorylated p38 (P38-P) with phospho-tau in a subpopulation of neurons and grains, suggesting the active participation of the kinase in tau phosphorylation, is observed as well in AGD (Ferrer et al., 2003, 2008) and in mice inoculated with AGD sarkosyl-insoluble fractions.

Similar deposits are reported following brain inoculation of AGD homogenates in WT mice with the exception of tau deposits categorized as TSAs (Clavaguera et al., 2013a, 2015).

A major differential point between AGD and AGD-inoculated mice is the presence of 3Rtau in addition to 4Rtau in tau-bearing neurons, oligodendrocytes, threads, and grains in AGD-inoculated mice. Since the AGD cases used for inoculation do not contain 3Rtau deposits, and the western blotting of sarkosyl-insoluble fractions shows a typical signature of 4Rtaupathy, the presence of 3Rtau implies the necessary phosphorylation of murine 3Rtau, in addition to 4Rtau, in the composition of abnormal tau deposits in mice inoculated with AGD sarkosyl-insoluble fractions.

For comparative purposes, another series of mice was inoculated with sarkosyl-insoluble fractions of PART, a primary 3R + 4Rtau neuronal-only tauopathy with NFTs in the temporal lobes (Crary et al., 2014). PART has also been considered part of AD without β-amyloid deposition and predominant early and middle stages of NFT pathology according to the classification of Braak and Braak of AD (Duyckaerts et al., 2015). Subtle differences in NFT pathology between PART, and early and middle stages of AD (Bell et al., 2019), are barely sufficient to firmly categorize these conditions as unrelated diseases (Jellinger, 2019). Inoculation of sarkosyl-insoluble fractions of PART results in the presence of 4Rtau and 3Rtau phospho-tau in neurons in the hippocampus, and of oligodendrocytes and threads in the corpus callosum three months after inoculation. Mice with 7 months of survival have an extension of tau deposits to distant regions such as the hypothalamus, and nuclei of the septum, and to the middle and contralateral corpus callosum, in addition to greater extension along the anterior-posterior axis when compared with mice inoculated with AGD at the same survival times. Despite the small number of animals used in the present study which precludes quantitative analysis, differences between AGD and PART may be due to several factors including different amounts of phospho-tau in the inoculums, different species of tau (as seen looking at the tau species in western blots of sarkosyl-insoluble fractions), and differing capacity of “fibril” formation (as illustrated by the different profiles of ThT amyloid quantification assay *in vitro*). Moreover, we cannot obviate the possibility that AGD spreading is slower than PART spreading as the longest survival time in both settings is 7 months.

Major differential points between PART and PART-inoculated mice are the presence of phospho-tau deposits in oligodendrocytes in inoculated mice which are absent in PART, and the lack of NFTs in mice in contrast with PART. Identification of NFTs was made by comparing the compact tangle morphology of tau deposits in PART neurons with the granular characteristics of neuronal tau deposits in mice. Here again, we cannot rule out the possibility that tangles in mice need more time than the maximal 7-month period available in the present study.

The present results are in line with the concept that abnormal tau has the capacity to seed and spread following the intracerebral inoculation of recombinant tau in transgenic mice expressing P301S or P301L tau mutation (Iba et al., 2013; Peeraer et al., 2015). Similarly, tau obtained from the brain of different human tauopathies has the capacity to seed and spread in transgenic mice expressing the longest human four-repeat tau isoform (ALZ17 line) (Clavaguera et al., 2013a, b), or in transgenic mice bearing the P301S tau mutation (line PS19) (Boluda et al., 2015). Neurons, oligodendrocytes and astrocytes, with particular features depending on the disease in question, are targets of tau seeding and spreading in all these settings, pointing to the importance of different tau strains in tauopathies. These observations are in agreement with other studies showing the variability of tau deposits depending on tau strains *in vitro* and *in vivo* (Sanders et al., 2014; Kaufman et al., 2016).

Pioneering inoculations of brain homogenates from human tauopathies in WT mice showed a limited variety of tau deposits in neurons and glial cells (Lasagna-Reeves et al., 2012; Clavaguera et al., 2013b). In another study, neurons were reported as the only targets of tau seeding and spreading following inoculation of AD homogenates (Guo et al., 2016). However, tau seeding and propagation in neurons, astrocytes and oligodendrocytes was observed following inoculation of paired helical filament (PHF)-enriched fractions from CBD and PSP; deposits in astrocytes comparable to astrocytic plaques were reported following inoculation of CBD, and doubtful tufted astrocytes following inoculation of PSP homogenates (Narasimhan et al., 2017). Finally, only grains, threads, and coiled bodies with phosphorylated tau were produced following inoculation of PHF-enriched fractions from AD in the dentate gyrus of WT mice a survival time of 3 months after inoculation (Audouard et al., 2016). These variations demonstrate different profiles of tau spreading depending not only on the disease but also on the methods and doses used for inoculation.

Tau can be transmitted trans-synaptically from one neuron to another neuron, based on connectivity rather than on proximity, thus facilitating progression of tau pathology in tauopathies (Liu et al., 2012; Ahmed et al., 2014; Dujardin et al., 2014b; Lewis and Dickson, 2016; Goedert and Spillantini, 2017; Goedert et al., 2017; Mudher et al., 2017; Gibbons et al., 2019). Tau secretion can also be produced by exo-synaptic secretory mechanisms, including free release of tau to the extracellular space, and vesicle-associated exocytosis. Free release to the extracellular space is indeed the means used to inoculate tau in the brain, and to make tau available to cultured cells. The vesicular-mediated secretary pathways, represented by microvesicles and exosomes, use the Endosomal Sorting Complex Required for Transport (ESCRT) (Abels and Breakefield, 2016). Microvesicles are generated by outward budding of the plasma membrane, whereas exosomes are rich in lipid rafts, and they are released upon depolarization of the plasma membrane (DeLeo and Ikezu, 2018; van Niel et al., 2018; Ruan and Ikezu, 2019). The two mechanisms may participate in tau secretion in different settings (Saman et al., 2012; Dujardin et al., 2014a; Lewis and Dickson, 2016; Goedert and Spillantini, 2017; Wang et al., 2017; DeLeo and Ikezu, 2018; Guix et al., 2018; Polanco et al., 2018; Ruan and Ikezu, 2019). Finally, tau can be transmitted through tunneling nanotubes which are actin-based nanotubular channels that connect one cell to another (Rustom et al., 2004; Davis and Sowinski, 2008). Tau is a component of nanotubes, and extracellular tau enhances the formation of nanotubes, and facilitates the transfer of tau from one cell to another (Tardivel et al., 2016). Tau uptake can be achieved through various mechanisms, including endocytosis, micropynocitosis, membrane fusion, and the activity of specific receptors (Frost et al., 2009; Kfoury et al., 2012; Holmes et al., 2013; Wu et al., 2013; Christianson and Belting, 2014; Mulcahy et al., 2014; Calafate et al., 2016; Bolós et al., 2017; Morozova et al., 2019).

Non-trans-synaptic transmission may be inferred as the process of tau uptake by oligodendrocytes following tau inoculation. Endogenous mouse tau is accumulated in oligodendrocytes in mice expressing transgenic human tau, thus indicating that mouse tau has the capacity to be recruited and aggregated in oligodendrocytes (Ren et al., 2014). Moreover, oligodendrocytes are the only cell targets, beside threads, following inoculation of sarkosyl-insoluble fractions from a wide variety of tauopathies in the corpus callosum of WT mice (Ferrer et al., 2018, 2019, 2020a,b). Similarly, human tau can be trapped by axons following inoculation. Then tau can be transported along nerve fibers and, more importantly, it can activate the phosphorylation of resident tau through the activation of kinases. In support of this, previous studies have shown that small misfolded tau is internalized via endocytosis, and it is anterogradely and retrogradely transported in neurons (Wu et al., 2013). Therefore, tau uptake by oligodendrocytes and axonal fibers is likely linked to endocytosis and micropynocitosis.

Differences between human AGD and AGD-inoculated mice are the result of multiple factors. Among these, the characteristics of tau are different in the two species. In adult human brain, 4Rtau and 3Rtau species are balanced, whereas in the adult mouse brain 4Rtau predominates. In addition to differences in the shift of isoform expression linked to exon 10 from embryonic to adult brain between murine and human brain, murine tau also differs from human tau in the N-terminal domain and three amino acid residues at the C-terminal domain (Goedert et al., 1988; Lee et al., 1988; Kampers et al., 1999; Goedert and Spillantini, 2019; Sayas et al., 2019). This is an important point as the N-terminal domain is crucial for specific protein-protein interactions (Stefanoska et al., 2018).

Moreover, the distribution and localization of the different tau isoforms in mice is region-dependent (Bullmann et al., 2007; McMillan et al., 2008; Liu and Götz, 2013). These differences may explain the distinct vulnerability and types of lesions between human diseases and inoculated WT mice which express only murine tau. Not only tau strains in the donor, but also tau strains in the host are critical to the formation of characteristic tau deposits. Moreover, differing cell vulnerability may depend on the availability of tau in particular cell types. Neurons and oligodendrocytes have a high capacity to recruit endogenous tau to form tau aggregates following inoculation of tau from different tauopathies; in contrast, the capacity of astrocytes is more limited. ARTAG homogenates produce tau seeding and spreading in neurons, oligodendrocytes and threads in WT mice, but astrocytes are rather resistant to producing tau inclusions following inoculation of ARTAG homogenates (Ferrer et al., 2018) and homogenates from other tauopathies in WT mice (Ferrer et al., 2019, 2020a,b), unless large amounts of tau are inoculated (Narasimhan et al., 2017).

Finally, an intriguing aspect is the deposits of 3Rtau following inoculation of sarkosyl-insoluble of AGD observed here and in other pure 4Rtau tauopathies (Ferrer et al., 2018, 2020a,b). It may appear that 3Rtau aggregates in inoculated mice are found in immature neurons which are recruited following the administration of tau. However, 3Rtau is not only found in neurons with mature appearance but also in oligodendrocytes and along the fibers of very long tracts such as the corpus callosum. In fact, little is known about the local machinery put in motion after intake of abnormal tau to recruit and actively phosphorylate local tau. Several regulatory elements exist in the introns flanking exon 10 during normal development, some upstream and others downstream, including inhibitors such as SRp30c, SRp55, SRp75, 9G8, U2AF, PTB, and hnRNPG, and activators such as htra2beta1, CELF3, and CELF4 (Gao et al., 2000; Sobrido et al., 2003; Wang et al., 2004; Andreadis, 2005). Nothing is known about the role of these regulatory factors following tau inoculation in murine models and in tau-treated cultured cells.

These results show the capacity for seeding and spreading of AGD tau in the brain of WT mice, and suggest that the characteristics of donor tau and host tau underlie common and specific aspects of deposits both in human disease and corresponding experimentally induced murine models. Understanding the mechanisms modulating tau recruitment and modifications in the host needs further efforts.

## Data Availability Statement

All datasets presented in this study are included in the article/supplementary material.

## Ethics Statement

The animal study was reviewed and approved by C.E.E.A: Comitè Ètic d’Experimentació Animal; University of Barcelona, Spain; ref. 426/18.

## Author Contributions

PA-B obtained the PHFs, inoculated the animals, and prepared the samples for histological studies. JS-J and VG assessed PHF using western blotting, thioflavin assays, and TEM. IF directed the experimental designed, studied human cases, studied the results in inoculated mice, and wrote the manuscript. JR contributed with the general idea of seeding and spreading of tauopathies, discussed the results through the experimental study. All authors contributed to the final version of the manuscript.

## Conflict of Interest

The authors declare that the research was conducted in the absence of any commercial or financial relationships that could be construed as a potential conflict of interest.
