# Corrigendum: Capacity for Seeding and Spreading of Argyrophilic Grain Disease in a Wild-Type Murine Model; Comparisons With Primary Age-Related Tauopathy

**DOI:** 10.3389/fnmol.2022.870475

**Published:** 2022-03-18

**Authors:** Isidro Ferrer, Pol Andrés-Benito, Julia Sala-Jarque, Vanessa Gil, José Antonio del Rio

**Affiliations:** ^1^Department of Pathology and Experimental Therapeutics, University of Barcelona, Barcelona, Spain; ^2^Bellvitge University Hospital, IDIBELL (Bellvitge Biomedical Research Centre), Barcelona, Spain; ^3^CIBERNED (Network Centre of Biomedical Research of Neurodegenerative Diseases), Institute of Health Carlos III, Ministry of Economy and Competitiveness, Madrid, Spain; ^4^Institute of Neurosciences, University of Barcelona, Barcelona, Spain; ^5^Molecular and Cellular Neurobiotechnology, Institute of Bioengineering of Catalonia (IBEC), Institute for Science and Technology, Parc Científic de Barcelona, Barcelona, Spain; ^6^Department of Cell Biology, Physiology and Immunology, Faculty of Biology, University of Barcelona, Barcelona, Spain

**Keywords:** argyrophilic grain disease, primary age-related tauopathy, tauopathies, tau, seeding, progression, coiled bodies

In the original article, there was a mistake in Figure 4 as published. Panels A, B, C, D, E, F of the published Figure 4 were incorrectly labeled. The corrected Figure 4 appears below.

**Figure 4:**
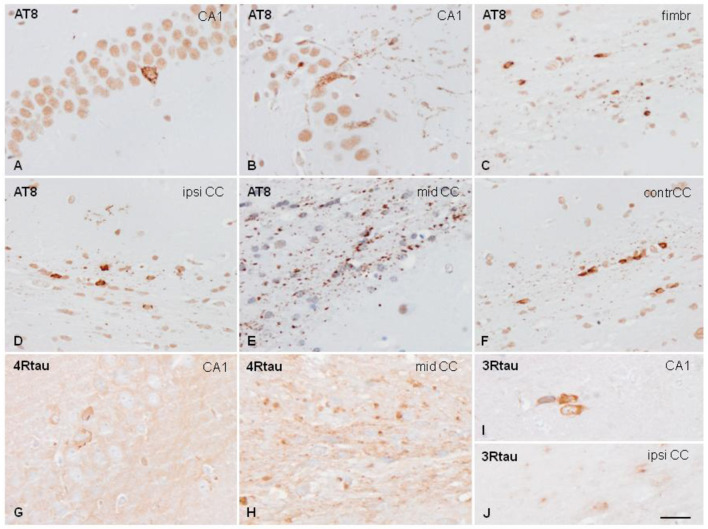
Hyper-phosphorylated tau-containing cells and threads following unilateral intra-hippocampal injection of sarkosyl-insoluble fractions from PART into WT mice at the age of 7 months and killed at the age of 10 months (3 months survival) **(A,C)**; 3 months and killed at the age of 10 months **(C,D–F)**; and at the age of 12 months and killed at the age of 19 months (7 months survival) **(G–J)**. Tau deposits in neurons, independently of the survival time, show granular deposits in the cytoplasm, and occasional denser inclusions with no similarities with tangles **(A,B)**. Threads and coiled bodies are abundant in the fimbria and corpus callosum **(C–F)**. Individual neurons, threads and oligodendrocytes in inoculated mice are stained with anti-4Rtau **(G,H)** and anti-3Rtau **(I,J)** antibodies. Paraffin sections slightly counterstained with hematoxylin. CA1, region of the hippocampus; fimbr, fimbria; ipsi contr CC, ipsi- and contralateral corpus callosum; **(A–F)**, bar = 50 μm; **(G–J)**, bar = 50 μm.

The authors apologize for this error and state that this does not change the scientific conclusions of the article in any way. The original article has been updated.

## Publisher's Note

All claims expressed in this article are solely those of the authors and do not necessarily represent those of their affiliated organizations, or those of the publisher, the editors and the reviewers. Any product that may be evaluated in this article, or claim that may be made by its manufacturer, is not guaranteed or endorsed by the publisher.

